# Risky sexual behavior and associated factors among university students in Ethiopia: a cross-sectional national survey

**DOI:** 10.1186/s12889-024-19213-2

**Published:** 2024-06-26

**Authors:** Wudinesh Belete Belihu, Minilik Demissie Amogne, Tobias Herder, Jesper Sundewall, Anette Agardh

**Affiliations:** 1 Social Medicine and Global Health, Lund University, Malmo, Sweden; 2 HIV/TB Research Directorate, Ethiopian Public Health Institute, Addis Ababa, Ethiopia

**Keywords:** Risky sexual behavior, University students, Multiple sexual partners, Condom use, Ethiopia

## Abstract

**Background:**

Risky sexual behavior (RSB) is one of the major youth sexual and reproductive health problems globally, including in Ethiopia. RSB among youth increases the risk of HIV infection, other sexually transmitted infections (STIs), unintended pregnancy, and unsafe abortion. Therefore, the aim of this study was to examine RSB and its associated factors among university students in Ethiopia.

**Methods:**

A cross-sectional study was employed in six randomly selected public universities in Ethiopia from August 2021 to February 2022. A stratified two-stage sampling technique was applied to reach the required number of study participants, and a structured self-administered questionnaire was used. RSB was defined as having had sexual relationships with more than one partner and using condoms with a new sexual partner irregularly or not at all in the last 12 months. Bivariable and multivariable logistic regression analyses were used to identify factors associated with RSB among those participants who were sexually active.

**Results:**

The prevalence of RSB among those who had had sexual intercourse in the last 12 months (*n* = 523) was 19.5% (*n* = 102). One hundred forty-four (29.9%) students had multiple sexual partners, and 325 (69.3%) students did not always use condoms with a new sexual partner. Adjusted odds ratios (AOR) showed that those students aged 21–24 years had lower odds of RSB than those aged above 25 years AOR 0.18 (95% CI 0.03–0.98). The adjusted odds of RSB were 6.7 times higher (95% CI 1.26–35.30) among students who started sex at the age of 10–17 years than those who started sex at 21 years and above and 3.9 times higher (95% CI 1.33–11.39) among students who had experienced emotional violence.

**Conclusion:**

RSB continues to be a problem among university students in Ethiopia. Those students who started sex at an early age and those who experienced emotional violence were more likely to engage in RSB. Therefore, universities in Ethiopia should implement strategies such as RSB targeted health education programs that consider early sexual debut, experiences of emotional violence, and safe sexual practices.

**Supplementary Information:**

The online version contains supplementary material available at 10.1186/s12889-024-19213-2.

## Background

Risky sexual behavior (RSB) is any sexual behavior that increases the likelihood of adverse sexual and reproductive health consequences, particularly among young people. Examples of such behaviors include unprotected sexual intercourse and having multiple sexual partners without consistent or correct condom use. The health consequences may include unintended pregnancy, unsafe abortion, HIV/AIDS, and other sexually transmitted infections (STIs) [1, 2].

According to the United Nations youth report 2020, there are 1.2 billion young people aged 15 to 24 years, accounting for 16 percent of the global population. The population growth rate remains high in sub-Saharan African countries with the youngest age distribution globally. For these countries as a group, the number of youth aged 15 to 24 is expected to rise from 207 million in 2019 to 336 million in 2050 [3]. In Ethiopia, young people aged 10–24 account for 35% of the total population [4].

Globally, in 2022, about 39.0 million people were living with HIV(PLHIV), and there were 1.3 million new HIV infections [5]. Of these, 65% of people PLHIV reside in sub-Saharan Africa. In Ethiopia, an estimated 2400 [1100–5800] new HIV infections occurred among young people aged 15–24 in 2022 [6]. As the youth population increases, unless sexual and reproductive health services also increase, the consequences of RSB will be a challenge. According to studies conducted at Ethiopian universities, the prevalence of RSB among sexually active students ranged from 30.2% to 44% [7–9]. However, RSB was variously defined across these studies. Participants who had sexual intercourse experience ever and engaged in at least one of the following behaviors were considered to have RSB: inconsistent use of condoms during sex, having multiple sexual partners, or early initiation of sex or sex with a commercial sex worker [7, 8] or having more than one sexual partner or inconsistent condom use during sex in a lifetime [9]. Among university students in Ethiopia, the prevalence of having more than one sexual partner ever and in the last 12 months was 61.1% [7] and 16.8% [10], respectively. Similarly, studies done among university students in Ethiopia showed that the prevalence of ever having used condoms irregularly or not at all ranged from 40.2% [7] to 81% [9]. Risk factors that have been associated with RSB among university students in Ethiopia and Kenya include early sexual debut, [10], having consumed alcohol [12], exposure to sexual violence as a child, [11], being male, [12, 13], and substance use among people of all ages regardless of their sex and occupation in Ethiopia [14].

The Sustainable Development Goals (SDG) aim to end the epidemic of AIDS, ensure universal access to sexual and reproductive healthcare services, ensure access to effective and essential medicine, and substantially reduce the proportion of youth not in employment, education, or training by 2030 [15]. Accordingly, the Ethiopian National Adolescent and Youth Health Strategy states that RSB is one of the major youth sexual and reproductive health problems in Ethiopia [16]. Currently, the student population at higher educational institutions is rapidly increasing [17], which might pose challenges with regard to students’ sexual and reproductive health. However, there is a limited number of comprehensive studies on the magnitude of risky sexual behavior (RSB) and its associated factors among university students in Ethiopia. Therefore, the overall objective of the study was to examine RSB and associated factors among university students in Ethiopia. Such knowledge may provide strategic information for strengthening efforts regarding HIV prevention and control and the sexual and reproductive health of students.

## Methods

### Study design and setting

We conducted a cross-sectional study at six randomly selected public universities in different parts of Ethiopia. These were Hawasa, Dire Dawa, Bahir Dar, Ambo, Addis Ababa, and Adama University.

The data collection was conducted from August 2021 to February 2022.

### Study population

The study population was undergraduate university students attending their second and third years of study. First-year students were excluded because they were new to the environment, and it was also difficult to measure their risk behavior during the last 12 months in the university setting. Fourth- and fifth-year students were excluded since most of the students in this year have completed their course of studies and leave the university compound for their apprenticeship.

### Sample size and sampling procedures

The sample size was determined using a population proportion sample size calculator for the various objectives. The calculated sample size was 493 for each university, considering a 45.4% prevalence of sexual violence [18], a 95% CI, a design effect of 1.2, and a non-response rate of 10%. This sample size was powered for each university, and the total sample needed for the six universities was 2958. However, during actual data collection, a total of 2988 students participated in the study because all students who volunteered in the selected departments were included.

A stratified two-stage sampling technique was applied to increase precision and representation among the required number of study participants. First, the universities were stratified as first- and second-generation, in order to examine whether the risk of RSB differed by the university’s year of establishment. First-generation universities such as Addis Ababa, Bahir Dar, and Hawasa University are older than second-generation universities such as Dire Dawa, Ambo, and Adama University. First-generation universities in Ethiopia differ from second-generation universities in terms of their settings, systems, and the establishment of services. The first-generation universities are located in larger towns and have a well-established university environment compared to second-generation universities. Such an environment can indeed create conditions that enable risky behaviors, such as alcohol consumption, drug use, and visiting nightclubs, etc. These factors might contribute to a higher likelihood of engaging in risky sexual behaviors among students. At the time of the study, there were nine first-generation and twelve second-generation universities in total. Three universities from each generation were randomly selected using the lottery method.

**Fig. 1:**
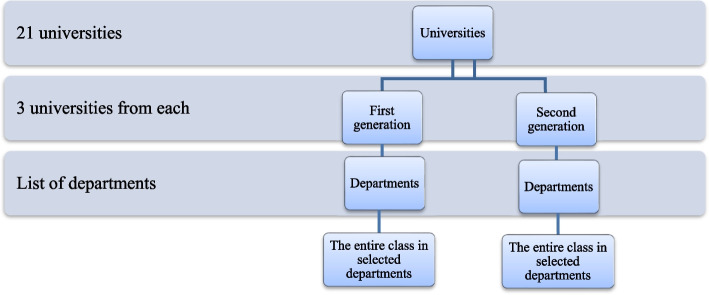
Sampling procedure among undergraduate university students

Secondly, at each university, on average 19 departments were selected by generating a random sample using Excel, based on the assumption that there was no difference between the departments concerning the students’ likelihood of engaging in RSB (Fig. 1). The lists of departments were obtained from the registrar of each university prior to the data collection. Then, all the students from the randomly selected second- and third-year classes in the selected departments were invited to participate. Students were informed about the study during their class sessions and asked for their voluntary participation. Finally, from those who volunteered to participate, written consent was collected, and they then completed a self-administered questionnaire in the classroom.

### Data collection tools

A structured self-administered questionnaire was used. The questionnaire included items about background characteristics, sexual behavior, HIV/AIDS and related questions, alcohol and drug use, reproductive health and exposure to violence. The questionnaire was adapted from a validated questionnaire about comprehensive knowledge of HIV using 27 items [19], the model AIDS Indicator Survey questionnaires developed by the Ethiopian Demographic and Health Survey [20, 21], a World Health Organization (WHO) indicator on alcohol consumption [22] and other similar studies [23–26]. The questionnaire was prepared in English and then translated into the local languages (Amharic and Oromifa) by professional language translators. It was pre-tested on 10% (*n* = 296) of the total sample size by students at one of the public universities. The inputs from the pre-testing were included in the final version of the questionnaire. Training was given to data collection coordinators about data collection procedures; close monitoring was conducted by the principal investigator.

HIV testing was performed for those students who volunteered, using the national testing algorithm. The testing was not mandatory to participate in the study.

### Variable definitions and measurement

#### Risky sexual behavior (dependent variable)

In the current analysis, RSB was a composite variable, defined as having both sexual relationships with more than one partner *and* using condoms with a new sexual partner irregularly or not at all in the last 12 months.

Having sexual relationships was defined as vaginal or anal or oral sexual intercourse, using the question “Have you had sex in the past 12 months?”. The responses were “Yes, vaginal sex”, “Yes, anal sex”, “Yes, oral sex”, and “No”. Those who affirmed at least one of the three “Yes” sexual practices were considered sexually active and those who chose “No” considered not sexually active in the past 12 months. Information about the number of partners was obtained using the following questions: “In the past 12 months, how many sexual partners have you had?”. This was dichotomized as having one sexual partner and having more than one sexual partner. Information about the frequency of condom use was obtained by inquiring “How often do you use a condom with a new sexual partner?”, with the response options “Always”, “Often”, “Sometimes”, and “Never”. Students who responded “Often”, “Sometimes”, and “Never” were considered irregularly or not at all using condoms.

#### Background variables (independent variables)

Age was an open-ended question that was categorized into three groups for the purpose of analysis: 18–20 years, 21–24 years, and > 25 years. Relationship status was dichotomized as “in a relationship” and “not in a relationship”. In a relationship means someone who has a boyfriend or girlfriend; it also includes those who were married and those living together. The monthly average expenditure was an open-ended question and then categorized into three groups, 1000 birr (< 20 $), 1001–2000 birr (20–40 $), and > 2000 birr (> 40 $), based on the current cost of living expenditure in the country.

Residence was categorized as urban vs rural. According to the Ethiopian Central Statistical Authority definition for surveys, urban residence is a locality with 2000 or more inhabitants and includes all administrative capitals of regions, zones, and woredas, as well as localities with at least 1000 people who are primarily engaged in non-agricultural activities and/or areas where the administrative official declares the locality to be urban, while rural residence comprises all areas not classified as urban. Information on the living status on campus was asked using the question, “Where do you live?”. The responses were “On campus” and “Off campus”. On campus means that students are living within the university campus, whereas off campus students are living outside the university. In addition, information on the living conditions while attending secondary school was obtained by inquiring “Did you mostly live at home or away from home while attending secondary school?”. The responses were “At home” and “Away from home”. At home means someone who was living with family while attending secondary school, whereas away from home means someone who was living away from his or her family while attending secondary school. Information was also obtained about the students’ sex, religion, year of study, and faculty of study.

#### HIV/AIDS knowledge, attitude towards HIV patients, and self-perception of acquiring HIV

The HIV/AIDS knowledge part of the questionnaire contained 13 questions (see Appendix). The range of knowledge scores was 0 to 13. Participants who scored at or above the mean (> 8) were considered to have a higher knowledge of HIV/AIDS, while those with scores below the mean were considered to have lower knowledge. To obtain the mean, the correct answers were coded as 1 and the incorrect answers as 0. Then, the total number of correct answers for 13 questions was summed for each participant. The mean score was calculated based on these totals. Similarly, attitude towards people living with HIV (PLHIV) was assessed using 8 questions, and the same method was used to obtain the mean. Those who scored at the mean and above (≥ 5.3) were considered to have a positive attitude, whereas those who scored below the mean were considered to have a negative attitude. To determine their self-perception of risk for acquiring HIV, participants were asked, “In your opinion, what is your risk of contracting HIV?”. The choices were “I am not at risk”, “I am at low risk”, “I am somewhat at risk”, “I am at high risk”, and “I am at very high risk”. Those who responded “I am somewhat at risk”, “I am at high risk”, and “I am at very high risk” were considered to be at risk of acquiring HIV.

#### Age at sexual debut and other sexual related variables

Age at sexual debut was an open-ended question and grouped into three categories: “10 to 17 years old”, “18 to 20 years old”, and “Older than or equal to 21 years old”. Additionally, information was obtained about having ever had sexual intercourse, and having had an HIV test.

#### Current use of contraceptive method

Information on the current use of contraceptive methods was asked using the question “Do you or your partner currently use a modern contraceptive method?”, with the response options “Yes” and “No”. Modern contraceptives include pills, intrauterine devices (IUD), injectables, condoms, Norplant, diaphragm, spermicides, tubal ligation, vasectomy, and rhythm method (sexual intercourse during a safe period). Students who were using one of the contraceptive methods and chose “Yes”, during the data collection period were considered as current use of contraceptives.

#### Alcohol consumption

Regarding alcohol consumption, heavy episodic drinking (HED) was defined as those who ever consumed five or more (for men) and four or more (for women) standard drinks of alcohol on at least one occasion in the past 30 days [28]. The question was “Have you ever consumed four/five or more standard drinks of alcohol on at least one occasion?”, with the response options “Never”, “Yes, before the last 12 months”, “Yes, in the last 12 months”, “Yes, in the last one month” and “Yes, in the last one week”. For the analysis, those who responded “Yes, in the last one month” and “Yes, in the last one week” were considered as “Yes” (HED), and the rest of the options were regarded as “No”. Additionally, the students were asked, “Have you ever consumed so much alcohol that you couldn’t remember what happened the next day?”. The response options were “I do not drink alcohol”, “I don’t remember”, “No, I didn’t consume so much alcohol”, “Yes, before the last 12 months”, and “Yes, in the last 12 months”. They were also asked about the frequency of alcohol consumption during the past month, using the question “In the past month, how often have you consumed alcohol?”, where the response options were “I do not drink alcohol”, “Less often than once every two weeks”, “Once every two weeks or more”, “Once a week or more”, “Every day” and “Other”.

#### Substance use variable

Substance use was assessed by the question, “Have you used any substance/ drugs/ intoxicants other than alcohol in the past 12 months?” and the options were “Khat”, “Ganja (Atsefaris)”, “Shisha”, “Hashish”, “Cocaine”, “Inhaling solvents such as benzine or glue”, “Marijuana (cannabis)”, “Never used”, and “Other specify”. Those who chose at least one of the options were categorized as “Yes”. Khat (Catha edulis) is a flowering stimulant plant containing the alkaloid cathinone, which causes excitement and euphoria [27]. Ganja is a colloquial term used to refer to cannabis, particularly the dried flowers and leaves of the Cannabis sativa plant. It is most commonly associated with its use as a psychoactive substance that contains tetrahydrocannabinol (THC), where it is typically smoked or ingested for its mind-altering effects [28]. Information on the frequency of substances/ drugs/ intoxicants use during the past month was asked, using the question “How often have you used the substances or intoxicants during the past month?”, The responses were, “Less often than once every two weeks”, “Once a week or more”, “Once every two weeks or more”, “Every day”, “I do not use drugs or intoxicant”, and “Other specify”.

#### Violence variables

Violence was assessed in terms of emotional, physical, and sexual violence. Emotional violence was assessed by the question, “Have you been exposed to any of the following threats or threats of violence in the past 12 months that were so dangerous or serious that they scared you?”. The response options were “Did anyone ever say or do something to humiliate you in front of others”, “Threaten to hurt or harm you or someone close to you”, “Insult you or make you feel bad about yourself”, “Other, specify”, and “No”. For the analysis, those who responded to at least one of the options except “No” were considered to have experienced emotional violence. Physical violence was assessed by the question, “Have you been a victim of any of the following physical violence at any time during the past 12 months?” where the response options were “Did anyone ever push you, shake you, or throw something at you”, “Slap you”, “Twist your arm or pull your hair”, “Punch you with his/her fist or with something that could hurt you”, “Kick you, drag you, or beat you up”, “Try to choke you or burn you on purpose”, “Threaten or attack you with a knife, gun, or any other weapon”, “Other, specify”, and “No”. For this study, those who responded to at least one of the options except “no” were considered to have experienced physical violence. Regarding sexual violence, this was assessed as follows, “In the past 12 months, have you been raped or forced to have sex against your will?” where the choices were “Yes” and “No”. Those who responded “Yes” were considered to have experienced sexual violence.

### Data analysis

Double entry using EpiInfo version 7.2.2.12 was performed by two independent data clerks in order to validate the consistency of the data and then exported to SPSS Version 26 for analysis. For the final analysis, the data collected from all six universities was aggregated into one data set. Descriptive statistics were used to provide summary measures such as frequency tables andpercentages. To identify factors associated with RSB, both bivariable and multivariable logistic regression analyses were performed. For the current regression analysis, only those who were candidates for RSB (those students who had sexual intercourse in the last 12 months, *n* = 523 participants) were included. Crude and adjusted odds ratios with 95% confidence intervals (CIs) were used to measure the strength of the associations. The level of significance p-value ≤ 0.25 was used to select candidate variables for the final multivariable analysis. The use of higher significance levels(< 0.25) helps to ensure important predictors are not excluded early on [29]. Additionally, a correlation analysis was conducted to assess the possibility of multicollinearity. A Correlation was detected between emotional violence and physical violence in the last 12 months (*r* = 0.82). Consequently, physical violence was removed from the regression analysis because the level of its significant association with RSB was lower than emotional violence. Statistical significance was accepted at the *p*-value of < 0.05. Cases with missing values were not included in the analysis.

### Ethical considerations

Ethical approval was obtained from the Scientific and Ethical Research Office of the Ethiopian Public Health Institute. Written informed consent was obtained from the study participants during their class sessions prior to administering the questionnaire. Individuals were informed that participation was voluntary and they could withdraw at any point in the study process. No personal identifier was collected and confidentiality of the information was maintained.

## Results

Table 1 shows the socio-demographic characteristics of the aggregated sample from the six universities. A total of 2988 (94.4%) students responded to the questionnaire among the total number of students in selected departments (*N* = 3165), and the proportion of those who tested for HIV was 81.9% among the total number of students in selected departments and 86.7% among those who completed the questionnaire. The majority of the participants, 68.7%, were between the ages of 21–24 years, and 65.1% were male. The majority of the participants (80.7%) were not in a relationship. In terms of their religion, 56.5% of the participants were Orthodox Christians. About three-fourths (73.0%) of the participants had urban residence before coming to the university, and 77.1% were living at home while attending secondary school. One-fourth of the participants were from the Faculty of Business and Economics and one-fourth from the Institute of Technology. Nearly all (92.8%) of them were living on campus, and half of the participants were second-year students. The majority of participants (66.8%) had a monthly average expenditure of < 1000 birr^1^).

**Table 1:** Socio-demographic characteristics and other related behavioral factors among undergraduate students at six Universities in Ethiopia, 2022 (*N* = 2988)

Variable	Frequency (N)	Percent (%)
**Age** (*n* = 2817)		
18—20 years	783	27.8
21—24 years	1936	68.7
> 25 years	98	3.5
Missing	171	
**Sex** (*n* = 2911)		
Female	1016	34.9
Male	1895	65.1
Missing	77	
**Relationship status** (*n* = 2841)		
In relationship	549	19.3
Not in relationship	2292	80.7
Missing	147	
**Religion** (*n* = 2911)		
Orthodox Christian	1645	56.5
Catholic	33	1.1
Protestant	744	25.6
Muslim	442	15.2
Other	47	1.6
Missing	77	
**Residence before coming to the university** (*n* = 2855)		
Urban	2085	73.0
Rural	770	27.0
Missing	133	
**Living conditions while attending secondary school** (*n* = 2893)		
At home	2230	77.1
Away from home	663	22.9
Missing	95	
**Generation of the university** (*n* = 2988)		
First generation	1497	50.1
Second generation	1491	49.9
**Faculty** (*n* = 2968)		
Faculty of Natural and Computational Science	539	18.1
Faculty of Medicine	48	1.6
Faculty of Social and Human Science	636	20.7
Faculty of Law	228	7.6
Faculty of Business and Economics	771	25.9
Faculty of Institute of Technology	746	25.0
Missing	20	
**Year of study** (*n* = 2988)		
Second year student	1467	49.1
Third year student	1521	50.9
**Living status on campus** (*n* = 2838)		
On campus	2635	92.8
Off campus	203	7.2
Missing	150	
**Monthly average expenditure** (*n* = 2025)		
< 1000 birr (< 20$)	1353	66.8
1001—2000 birr (20$-40$)	467	23.1
> 2000 birr (> 40$)	205	10.1
Missing	963	

Cases with missing data were not included in the analysis

Table 2 shows sexual and other related behavioral factors among undergraduate students at six Universities in Ethiopia.

**Table 2:** Sexual and other related behavioral factors among undergraduate students at six Universities in Ethiopia, 2022 (*N* = 2988)

Variable	Frequency (N)	Percent (%)
**Ever had sexual intercourse** (*n* = 2778)		
Yes	771	27.8
No	2007	72.2
Missing	210	
**Sexually active in the past year** (*n* = 755)		
Yes	523	69.3
No	232	30.7
Missing	16	
**Frequency of condom use with a new sexual partner** (*n* = 691)		
Never	269	38.9
Sometimes	152	22.0
Often	104	15.1
Always	166	24.0
Missing	80	
**Frequency of condom use in the last 12 months** (*n* = 469)		
Never	129	27.5
Sometimes	109	23.2
Often	87	18.6
Always	144	30.7
Missing	54	
**Number of sexual partner/s in the last 12 months** (*n* = 481)		
One	337	70.1
More than one	144	29.9
Missing	42	
**Currently used contraceptive** (*n* = 457)		
Yes	211	46.2
No	246	53.8
**HIV test result** (*n* = 2592)		
Positive	5	0.2
Negative	2,587	99.8
**HIV test result among RSB** (*n* = 523)		
Positive	3	0.57
Negative	520	99.43
**RSB in the last 12 months** (*n* = 523)		
Yes	102	19.5
No	421	80.5

Cases with missing data were not included in the analysis

Regarding sexual behavior, 27.8% of the study participants had ever had sexual intercourse and of these, 69.3% were sexually active in the past year. Among those who had ever had sex, only 24.0% of them always used condoms. In the last 12 months, 30.7% of the participants used condoms always, 29.9% had more than one sexual partner and 46.2% used contraceptives.. Furthermore, in this study, HIV testing was conducted and 5(0.2%) of the students were HIV positive among all study participants and 3 (0.57%) among those who engaged in risky sexual behavior in the last 12 months.

### Risky sexual behavior

A total of 523 students were sexually active during the past year. Of those who responded to the question about number of sexual partners (*n* = 481), 144 (29.9%) had more than one partner, and of those who responded to the frequency of condom use (*n* = 469) in the past year, 325 (69.3%) did not always use a condom. The RSB prevalence was 19.5% (*n* = 102) (both having more than one sexual partner and not using a condom always among those who had sexual intercourse in the last 12 months) and 3.4% among the total study participants (*n* = 2988).

### Factors associated with risky sexual behavior

Table 3 shows the results of the bivariable and multivariable logistic regression analyses concerning factors associated with RSB. The model includes 523 study participants who were eligible for RSB in the last 12 months. The selection of the reference category was based on the assumption of low risk associated with RSB.

**Table 3:** Bivariable and multivariable logistic regression analysis of factors associated with risky sexual behavior among undergraduate students at six universities in Ethiopia, 2022 (*n* = 523)

Variables	Total	COR (95% CI)	*p*-value	AOR (95% CI)	*P*-value
**Age**					
18—20 years	92	1.77 (0.69–4.49)	0.234*	0.29 (0.04–1.90)	0.195
21—24 years	361	1.08 (0.46–2.53)	0.864	0.18 (0.03–0.98)	0.047**
> 25 years	42	1		1	
**Sex**					
Female	144	1			
Male	362	1.29 (0.78–2.12)	0.323		
**Relationship status**					
Married/in a relationship	208	1			
Not in a relationship	285	1.27 (0.81–2.02)	0.300		
**Religion**					
Orthodox Christian	253	1			
Catholic	7	1.95 (0.37–10.40)	0.433		
Protestant	143	1.24 (0.74–2.10)	0.416		
Muslim	87	1.27 (0.69–2.35)	0.439		
Other	15	1.78 (0.54–5.84)	0.344		
**Generation of the university**					
First	231	0.95 (0.61–1.47)	0.815		
Second	292	1			
**Year of study**					
Second year student	227	1		1	
Third year student	296	1.69 (1.07–2.67)	0.023*	2.68 (1.0–7.25)	0.052
**Living status in campus**					
On campus	435	1	0.918		
Off campus	56	0.96 (0.48–1.94)			
**Residence before coming to the university**					
Rural	130	1	0.925		
Urban	367	0.98 (0.59–1.61)			
**Monthly average expenditure**					
< 1000 birr (< 20$)	175	1		1	
1001—2000 birr (20$-40$)	109	1.64 (0.91–2.99)	0.103	2.03 (0.73–5.69)	0.177
> 2000 birr (> 40$)	47	2.23 (1.06–4.69)	0.035*	1.16 (0.30–4.52)	0.835
**Age at the start of sex**					
10 to 17 years	129	2.85 (1.33–6.13)	0.007*	6.68 (1.26–35.30)	0.025**
18 to 20 years	244	1.08 (0.51–2.30)	0.833	1.443 (0.29–7.11)	0.652
21 and above years	67	1		1	
**Ever consumed so much alcohol that you couldn’t remember what happened the next day**					
I do not drink alcohol	256	1		1	
I don’t remember	87	0.59 (0.17–2.05)	0.408	1.42 (0.216–9.33)	0.716
No, didn’t consume so much alcohol	38	1.13 (0.62–2.07)	0.692	0.74 (0.18 -3.13)	0.683
Yes, before last 12 months	91	0.51 (0.17–1.50)	0.223*	0.18 (0.02–1.99)	0.162
Yes, in the last 12 months	25	1.30 (0.73–2.32)	0.375	1.60 (0.55–4.70)	0.391
**Frequency of drinking alcohol in the past month**					
Less often than once every two weeks	70	0.81 (0.40–1.60)	0.537		
Once every two weeks or more	53	1.02 (0.49–2.11)	0.959		
Once a week or more	43	0.76 (0.32–1.79)	0.526		
Every day	18	0.49 (0.11–2.18)	0.346		
I do not drink alcohol	269	1			
**Heavy episodic drinking in the past one month**					
Yes	69	0.90 (0.46–1.76)	0.764		
No	407	1			
**Chewing khat (*n* = 491)**					
Yes	78	1.80 (1.04–3.13)	0.037*	1.26 (0.29–5.41)	0.758
No	413	1			
**Frequency of substance used in the past month**					
Less often than once every two weeks	42	1.40 (0.65–3.00)	0.385	0.84 (0.18 -3.98)	0.829
Once a week or more,	39	1.34 (0.61–2.98)	0.464	1.01 (0.19–5.25)	0.989
Once every two weeks or more,	33	1.43 (0.62–3.33)	0.401	0.93 (0.146–5.90)	0.938
Every day	21	0.22 (0.03–1.70)	0.148*	0.36 (0.03–5.32)	0.459
I do not use drugs or intoxicant	340	1			
**Emotional violence experience in the last 12 months**					
Yes	166	1.42 (0.90–2.24)	0.128*	3.89 (1.33–11.39)	0.013 **
No	338	1		3.89 (1.33–11.39)	0.013 **
**Raped/forced sex experience in the last 12 months**					
Yes	46	1.63 (0.82–3.25)	0.161*	0.86 (0.19–4.00)	0.85
No	417	1			
**Self-perception on the risk of acquiring HIV**					
Perceived at risk	173	2.21 (1.41–3.44)	0.001*	1.81 (0.713–4.59)	0.212
Not perceived at risk	328	1			
**HIV/AIDS knowledge**					
Higher	310	1			
Lower	191	1.99 (1.28–3.12)	.002*	1.18 (0.45–3.07)	0.742
**Currently used contraceptive**					
Yes	211	1			
No	246	2.72 (1.65–4.47)	< 0.001*	1.95 (0.77–4.94)	0.157

*COR* Crude Odd Ratio, *AOR* Adjusted Odd Ratio, *CI* Confidence interval, *1* Reference category

^*^P-value < 0.25 was used for multivariable analysis; ** *P*-value < 0.05

The results of the bivariable analysis showed that younger age (18–20 years), being a third-year student, having a monthly average expense > 2000 birr (> 40$), age at which sex started (10–17 years), having consumed so much alcohol before the last 12 months, chewing khat, substance use every day, experienced emotional violence in the last 12 months, experienced sexual violence in the last 12 months, perceived being at risk of acquiring HIV, having lower HIV/AIDS knowledge and using contraceptive currently were candidates for inclusion in the multivariable analysis (crude odds ratio, *p*-value < 0.25).

In the fully adjusted model, where all variables were mutually adjusted for one another, multivariable analysis showed that RSB was significantly associated with age (21–24 years), age at the start of sex (10–17 years), and having experienced emotional violence in the last 12 months. Adjusted odds ratios (AOR) showed that students aged 21–24 years had lower odds (0.18) (95% CI 0.03–0.98) of RSB than those aged above 25 years. The adjusted odds of RSB were 6.7 times higher (95% CI 1.26–35.30) among those students who started sex at the age of 10–17 years compared to those 21 and older. Furthermore, the adjusted odds of RSB were 3.9 times higher (95% CI 1.33–11.39) among students who had experienced emotional violence compared to those without such experience.

## Discussion

This nationwide study examined the prevalence of RSB and associated factors among university students in Ethiopia. The study found an overall prevalence of RSB 3.4% among all the study participants and 19.5% among those who had sexual intercourse in the last 12 months. Students aged 21–24 years were less likely to engage in RSB than students aged 25 years and above. Higher odds of RSB were seen among students who began sexual intercourse between the ages of 10 and 17 years and who had suffered emotional violence within the previous 12 months.

The prevalence of RSB in our study was lower than in the previous studies conducted at universities in Ethiopia, (30.2%) [7], (31.4%) [8], and (44%) [9]. This might be due to the difference in the operational definition of RSB. Most of the studies defined students with RSB as those who fulfilled at least one of the following behaviors: having multiple sexual partners, irregular or never used condom, starting sexual intercourse before 18 years of age, being forced to have sexual intercourse, or having sexual intercourse with a sex worker, whereas in this study, only those who both had multiple sexual partners and irregular/never usage of condom were considered to have RSB. The more stringent definition used in the current study can make it easier to identify those people who are most prone to engage in RSB and/or whose RSB might be especially worrisome, in that people who have multiple sexual partners and who are not regularly using condoms are at high risk for adverse health consequences, such as STIs including HIV, and unwanted pregnancy [2]. The current definition can also help to make interventions more effective by targeting the people who might most need RSB prevention strategies and who are also most likely to benefit, thus enabling an effective allocation of resources in settings where resources might be limited, such as in Ethiopia. In addition, the lower RSB prevalence currently obtained might be due to the COVID-19 pandemic, as movement was restricted and universities were closed for some months before the data collection period, which might have led to reduced sexual activity. A study about sexual intercourse and relationships before and during the COVID-19 pandemic among undergraduate students in the U.S. revealed that the COVID-19 pandemic and campus closure disrupted college students’ relationships and partnered sexual lives [30].

Even though the current prevalence of RSB obtained was lower than in previous studies conducted at Ethiopian universities [7–9], the rates of RSB obtained merit prevention strategies. Creating awareness using peer educators about sexual health already in the first year of university and/or before that with particular emphasis on the consequences of RSB as well as the need for its prevention may help to reduce the incidence of risky sexual behavior. A study conducted in Ethiopia on the effects of peer education interventions on HIV/AIDS related sexual behaviors among secondary school students showed that the intervention group was more likely to use condom consistently in the last 12 months compared to students in the control group [31].

Several factors might contribute to university students engaging in risky sexual behaviors, such as being in a new environment, being away from their parents, and making independent decisions for the first time. Students may also be under a great deal of stress and may be more likely to be influenced by peer pressure. A study conducted among high school students in Rwanda showed that students who were under peer pressure influence were about four times more likely to engage in RSB than those who were not [32]. Additionally, some university students may have low self-esteem, which can make them more likely to engage in risky sexual behaviors in order to feel better about themselves. A study examining relationships between sexual history, self-esteem, and emotional distress, and their impact on future sexual risk behavior among sexually active adolescent females in US revealed that adolescent females with lower self-esteem were more likely to have unprotected sex [33].

One of the factors significantly associated with RSB was age, where students aged 25 and above years were more likely to engage in RSB than those aged 21–24 years. This finding is similar to studies conducted among university students and youth center reproductive health clinic users and non-users in Ethiopia [9, 34] and among ethnically diverse university students in the US [35]. This might be due to the likelihood of risky behaviors such as alcohol consumption and drug use being higher among older university students [36–38]. Hence, age-specific targeted interventions for students already engaged in RSB, along with prevention strategies for those not yet involved in RSB through educational programs, could be crucial.

Another factor significantly associated with RSB was the age at sexual debut, where students who started sex at the age of 10–17 years had higher odds of RSB. This finding is in line with a study conducted among students in Ethiopia [10], a population-based cross-sectional study conducted in Zambia among sexually active female adolescents aged 15–19 years [39], and a cross-sectional study in China among college students, where the odds of having condom-less sex at the latest sexual intercourse increased among those who reported early sexual debut [40]. A study conducted on sexual behavior among university students in China showed that those who started sex at an early age might have more sexual partners in their lifetime than those who started sex late [41]. The association between early initiation of sexual intercourse and RSB could be due to the longer duration of their sexual experience and a lower likelihood of adopting preventive behaviors [10, 41]. Further, it could also be that those who engage in early sexual activity may normalize RSB compared to their peer groups. Therefore, a comprehensive sexuality education targeting both those who have and who have not started sex and that focuses on safer sexual practices, including abstinence and appropriate condom use, is recommended. A study conducted on the outcome of a comprehensive sexuality education initiative for delaying sexual debut among adolescents in public secondary schools in Mexico revealed that students who did not receive the initiative (comparison schools) were 4.7 times more likely to start sexual activity than students who received comprehensive sexuality education in the intervention schools [42].

According to WHO, exposure to violence including emotional violence can result in psychological distress that can lead an individual to engage in risky practices like drinking alcohol, using intoxicating drugs, or having unprotected sex as a coping mechanism [43]. In the current study, the odds of RSB were higher among students who had experienced emotional violence than those with no such experience. A study conducted among high school students in Rwanda [32] found that RSB was significantly associated with domestic violence, and a study conducted among university students from 25 countries in Africa, the Americas and Asia [44] revealed that exposure to emotional violence was significantly associated with addictive and other risky behaviors. Moreover, emotional abuse can decrease a person’s self-esteem, making them feel worthless [45]. Studies conducted on self-esteem and risky sexual behavior among adolescents in the U.S and Czech Republic revealed that adolescents with low self-esteem were more likely to engage in risky sexual behavior [33, 46]. Thus, capacity building on self-esteem and confidence, education on emotional violence prevention and management, and creating a support mechanism from friends, family, or counselor are suggested strategies for addressing this problem.

In this study, factors such as being in the third year of study, having a monthly average expenditure > 2000 birr (> 40 $), heavy episodic drinking in the past month and chewing Khat were associated with RSB in the bivariable analyses but the associations could not be established in the multivariable analysis. However, other similar studies conducted among undergraduate university students in Ethiopia, [10], [8], and [9], reported that those students who drank alcohol had significantly higher odds of RSB than those who did not drink alcohol. Studies conducted in Ethiopia [7], [8], and [10] found that Khat chewing was significantly associated with RSB.

### Methodological considerations

This study has a number of strengths. To our knowledge, this is the first national survey in Ethiopia that involved six universities, while other studies were limited to one university. The study is thus nationally representative of all undergraduate university students in Ethiopia, with the possible exception of first-year students who were intentionally excluded. The results may have limited generalizability to university students in other countries due to variations in cultural and sociodemographic factors. The HIV testing of students was done using the national testing algorithm. An additional strength is the pilot study that was done before the actual data collection, which ensured that the questionnaire items were appropriate for the target group. Administering the questionnaire in the classroom could have increased participation in the study. The comprehensive nature of the questionnaire is an additional strength.

The study also has some limitations. Since the questionnaire was self-administered, there might be incomplete or inaccurate data, as well as underreporting and overreporting of data due to different factors. To minimize these problems, orientation about how to complete the questionnaire was provided before administering the questionnaire. Another limitation is the possibility of social desirability bias due to the sensitive nature of some of the questionnaire items. However, the anonymous nature of study participation helped to reduce such bias. Moreover, to minimize the bias, after the questionnaire was completed, the students left it in the box that was prepared for questionnaire collection and the data collection facilitators were persons external to the universities. Furthermore, due to the cross-sectional study design, it is not possible to determine the cause-and-effect relationship between risk factors and the outcome variable (RSB). In this study, the stringent definition of RSB utilized might have limited the identification of students engaging in any type of RSB, yet it may allow for more effective interventions targeting those at high risk. There were some questionnaire items with missing data, but no systematic pattern could be observed, and therefore, the distribution of the missing data was deemed to be random.

## Conclusion

RSB continues to put university students at risk of adverse sexual and reproductive health outcomes. Age, early sexual debut and experience of emotional violence were identified as the factors associated with RSB. Universities in Ethiopia, in collaboration with the Ministry of Health and partners working on sexual and reproductive health, may need to develop or strengthen strategies to prevent factors that make youth more likely to engage in risky sexual behavior and reduce its consequences among university students. Providing age appropriate comprehensive sexuality education in schools could contribute to preventing RSB among adults.

### Supplementary Information

Supplementary Material 1.

## Data Availability

The study data is available at the Ethiopian Public Health Institute on reasonable request using the following address. Ethiopian Public Health Institute. Swaziland street (The former Arbegnoch street), Gulele sub-city, Addis Ababa. P. O. Box 1242/5654.

## Abbreviations

AIDS: Acquired Immunodeficiency Syndrome
AOR: Adjusted Odds Ratio
CI: Confidence Interval
COR: Crude Odds Ratio
EPHI: Ethiopian Public Health Institute
HED: Heavy Episodic Drinking
HIV: Human Immunodeficiency Virus
PLHIV: People Living with HIV
RSB: Risky Sexual Behavior
SDG: Sustainable Development Goal
STI: Sexually transmitted infection
WHO: World Health Organization

## Footnotes

1 The average exchange rate for 1 United States dollar during the time of data collection was 52 Ethiopian birr.
