# Supplementary material for: Risky sexual behavior and associated factors among university students in Ethiopia: a cross-sectional national survey
Source: BMC Public Health. 2024 Jun 26;24:1701. doi: 10.1186/s12889-024-19213-2 (PMC11201902; doi:10.1186/s12889-024-19213-2)
Supplement: Supplementary file 1 — Supplementary Material 1. [file 12889_2024_19213_MOESM1_ESM.docx]

**Appendix**

**Section 2: Knowledge on HIV/AIDS**

| **Q#** | **Questions** | **Coding Categories** | **Skip to** |
| --- | --- | --- | --- |
| 1. | Have you heard of HIV or AIDS? | 1. Yes 2. No | If your answer is  "No", go to Section 4 Q# 1 |
| 2. | If yes, from where did you get the information about HIV/AIDS?  **(more than one response is possible)** | 1. My parents 6. Religious leaders 2. Sexual partner 7. Newspapers, posters or   pamphlets   1. Friends 8. Radio 2. Health institution 9. Your university 3. Teachers 10. Others, specify |  |
| 3. | Can a person reduce their risk of getting HIV by using a condom every time they  have sex? | 1. Yes 8. I don’t know 2. No |  |
| 4. | Can a person reduce their risk of getting  HIV by limiting sexual intercourse to one uninfected partner? | 1. Yes 8. I don’t know 2. No |  |
| 5. | Is it possible for a healthy-looking person  to have HIV? | 1. Yes 8. I don’t know 2. No |  |
| 6. | Can a person get HIV from a mosquito  bite? | 1. Yes 8. I don’t know 2. No |  |
| 7. | Can a person get HIV by sharing food with  a person who has HIV? | 1. Yes 8. I don’t know 2. No |  |
| 8. | Can HIV be transmitted from a mother to her baby: During pregnancy?  During delivery? By breast feeding?  (more than one response is possible) | Yes No I don’t know  During pregnancy----1 2 8  During delivery------ 1 2 8  By breast feeding----1 2 8 |  |
| 9. | Can the risk of mother to child transmission be reduced by the mother  taking antiretroviral (ARVs) drugs? | 1. Yes 8. I don’t know 2. No |  |
| 10. | Can a person get HIV through injections with a needle that has been used by  someone who has HIV? | 1. Yes 8. I don’t know 2. No |  |
| 11. | Can a person get HIV by shaking  hands with someone who has HIV? | 1. Yes 8. I don’t know 2. No |  |
| 12. | Can HIV be cured? | 1. Yes 8. I don’t know 2. No |  |
| 13. | Can treatment (antiretroviral) lower the  risk of HIV transmission from a person living with HIV? | 1. Yes 8. I don’t know   No |  |

**Section 3: Attitude towards HIV/AIDS**

**Now I would like to ask you some questions about your attitude towards HIV/AIDS transmission. Please remember that your response is confidential, and be truthful in your answers.**

| **Q#** | **Questions** | **Categories** | |
| --- | --- | --- | --- |
| 1. | If one of your family members was infected with HIV, would you be willing to care for him/her? | 1. Yes | 2. No |
| 2. | If you were infected with HIV, would you prefer this information to remain a secret? | 1. Yes | 2. No |
| 3. | Would you eat from the same plate as a person you know has HIV/AIDS? | 1. Yes | 2. No |
| 4. | Would you attend the same class at school/university as someone who you know has HIV? | 1. Yes | 2. No |
| 5. | If a professor has HIV, should he/she be allowed to continue to teach at the school/university? | 1. Yes | 2. No |
| 6. | If a health worker (doctor, nurse, etc) has HIV should he/she be allowed to continue to work with patients? | 1. Yes | 2. No |
| 7. | If you know a food seller who has HIV, would you buy from him/her? | 1. Yes | 2. No |
| 8. | Should students infected with HIV/AIDS have separate washing and toilet facilities at  school/university? | 1. Yes | 2. No |
